# Altered oral microbiome composition in mental disorders: a systematic review and meta-analysis

**DOI:** 10.1080/20002297.2025.2541828

**Published:** 2025-08-03

**Authors:** Dingxin Cao, Jun Yang, Yiwen He, Xinkang Zheng, Yanan Li, Yadong Chen, Yan Tu

**Affiliations:** a Department of Endodontics, Stomatology Hospital, School of Stomatology, Zhejiang University School of Medicine, Zhejiang Provincial Clinical Research Center for Oral Diseases, Key Laboratory of Oral Biomedical Research of Zhejiang Province, Cancer Center of Zhejiang University, Engineering Research Center of Oral Biomaterials and Devices of Zhejiang Province, Hangzhou, China; b Department of Oral Preventive Medicine, Shaoxing Stomatological Hospital, Shaoxing, China

**Keywords:** Oral microbiota, mental disorders, microbiome dysregulation, alpha diversity, salivary biomarkers, autism spectrum disorder

## Abstract

**Introduction:**

Emerging research underscores the gut-brain axis in mental disorder pathophysiology, yet the oral microbiome's contribution to mental health remains underexplored. Elucidating oral microbial signatures in mental and neurological disorders may reveal novel pathobiological mechanisms and advance biomarker discovery for precision diagnostics and microbiota-targeted interventions.

**Methods:**

This systematic review and meta-analysis investigates oral microbiota alterations across 6 different mental disorders, by synthesizing data from 20 case-control studies retrieved from PubMed, Embase, and Cochrane Library. Relative microbial abundance and beta diversity indices were extracted from between-group comparisons. Random-effects meta-analyses were conducted for alpha diversity to characterize microbiota differences between patients and controls.

**Results:**

Key findings included a significantly higher Simpson Index in patients (SMD = 0.42; 95% CI, 0.25 to 0.60) compared to controls. Beta diversity varied significantly only in SZ and MDD. Condition-specific variations in microbial abundance were observed: *Rothia* enrichment in ASD, overrepresentation of H_2_S-producing genera in SZ, and reduced *Solobacterium* and *Leptotrichia* in MDD.

**Conclusion:**

Collectively, the meta-analytical synthesis suggests alterations in oral microbiota diversity across mental disorders. Disease-associated microbial shifts highlight the oral microbiome as a candidate factor warranting further investigation for potential diagnostic applications and microbial-targeted therapeutic strategies.

## Introduction

Mental disorders – including depression, anxiety, bipolar disorder, schizophrenia (SZ), autism spectrum disorder (ASD), and substance use disorders – represent chronic conditions marked by significant comorbidity and suboptimal treatment responses [1–3], These disorders create substantial societal burdens, responsible for 418 million disability-adjusted life years (DALYs) globally in 2019 (16% of total DALYs) – a threefold increase from previous estimates [4]. Analysis of the 2019 Global Burden of Disease data revealed a 13.0% global prevalence of mental disorders, with substance use disorders (2.2%) dominated by alcohol use disorder (AUD)(1.5%) [5].

Extensive evidence highlights the critical role of gut microbiota in mental disorders [6,7]: Torres-Fuentes et al. demonstrated how gut microbiota regulates host metabolism, appetite, and brain reward systems, contributing to metabolic and neurological dysregulation in conditions like obesity and bulimia nervosa [8]; Similarly, Góralczyk-Bińkowska et al. linked gut microbial alterations to depression, SZ, bipolar disorder, ASD, and attention-deficit/hyperactivity disorder, noting distinct microbial profiles in affected individuals [9]. This bidirectional communication between gut microbiota and brain function – mediated by metabolic, endocrine, neural, and immune pathways – is termed the microbiota – gut – brain axis. Influenced by factors such as stress, diet, birth mode, probiotics, circadian rhythms, and environmental exposures, this axis plays a pivotal role in modulating neurological and behavioral outcomes [9]. These findings have spurred efforts to identify gut microbiota-based biomarkers – defined as ‘a substance, structure, or process that can be measured in vivo to assess or predict the onset, progression, or therapeutic response of a disease’ – with the goal of improving diagnostic accuracy, guiding targeted therapies, and monitoring treatment outcomes [10].

Substantial evidence has accumulated regarding gut microbiota alterations in various mental disorders, with microbial signatures being proposed as potential biomarkers. However, no studies have yet attempted to evaluate condition-specific alterations of oral microbiota across mental disorders – despite being the body’s second-largest microbial community after the gut [11]. Given the clinical advantages of oral sampling accessibility and recent advancements in 16S rRNA sequencing technology, oral microbiota may offer superior diagnostic potential [12]. Therefore, identifying disease-associated, condition-specific alterations in oral microbiota among patients with particular mental disorders could significantly advance biomarker discovery.

Our investigation addresses this critical gap through a systematic review and meta-analysis of oral microbiota alterations across six mental disorders: ASD, SZ, major depressive disorder (MDD), AUD, obsessive-compulsive disorder (OCD), and panic disorder (PD). This study evaluates the specificity and reproducibility of oral microbiota alterations in these mental disorders, with the goal of identifying potential microbial biomarkers”.

## Methods

The study protocol was prospectively registered on PROSPERO (CRD42024618295). We adhered to the Preferred Reporting Items for Systematic Reviews and Meta-Analyses (PRISMA) guidelines [13] and Cochrane recommendations for updated reviews [14].

### Search strategy

On 18 November 2024, we systematically searched PubMed, Embase, and the Cochrane Library for original studies using a combination of MeSH terms and free-text keywords. For PubMed, the search strategy included terms such as:

(Mental Disorders) OR (Mental Disorder) OR (Mental Illness) OR (Illness, Mental) OR (Mental Illnesses) OR (Psychiatric Disorders) OR (Psychiatric Disorder) OR (Psychiatric Diseases) OR (Psychiatric Disease) OR (Psychiatric Illness) OR (Psychiatric Illnesses) OR (Behavior Disorders) OR (Diagnosis, Psychiatric) OR (Psychiatric Diagnosis) OR (Mental Disorders, Severe) OR (Mental Disorder, Severe) OR (Severe Mental Disorder) OR (Severe Mental Disorders) OR (Schizophrenia) OR (Schizophrenias) OR (Dementia Praecox) OR (Schizophrenic Disorders) OR (Disorder, Schizophrenic) OR (Disorders, Schizophrenic) OR (Schizophrenic Disorder) OR (Autism Spectrum Disorder) OR (Autistic Spectrum Disorder) OR (Autistic Spectrum Disorders) OR (Disorder, Autistic Spectrum) OR (Autism Spectrum Disorders) OR (Depressive Disorders, Major) OR (Major Depressive Disorders) OR (Major Depressive Disorder) OR (Depression, Involutional) OR (Involutional Depression) OR (Melancholia, Involutional) OR (Involutional Melancholia) OR (Psychosis, Involutional) OR (Involutional Psychoses) OR (Involutional Psychosis) OR (Psychoses, Involutional) OR (Paraphrenia, Involutional) OR (Involutional Paraphrenia) OR (Involutional Paraphrenias) OR (Paraphrenias, Involutional)
(Oral) OR (Saliva) OR (Salivas) OR (Mouth) OR (Cavitas Oris) OR (Oral Cavity) OR (Cavity, Oral) OR (Vestibule of the Mouth) OR (Vestibule Oris) OR (Oral Cavity Proper) OR (Cavitas oris propria) OR (Mouth Cavity Proper) OR (Oropharynx)
(Microbiota) OR (Microbiotas) OR (Microbial Community) OR (Community, Microbial) OR (Microbial Communities) OR (Microbial Community Composition) OR (Community Composition, Microbial) OR (Composition, Microbial Community) OR (Microbial Community Compositions) OR (Microbiome) OR (Microbiomes) OR (Human Microbiome) OR (Human Microbiomes) OR (Microbiome, Human) OR (Microbial Community Structure) OR (Community Structure, Microbial) OR (Microbial Community Structures) OR (Oral Microbiota)
Searches 1 and 2 and 3 were then be combined (1 ‘AND’ 2 ‘AND’ 3)

The search strategy was adapted for EMBASE and the Cochrane Library. Additional search terms were identified using descriptive terms under MeSH headings. The search was limited to original human studies published in English from the year 2000 onward.

### Selection criteria

Studies were eligible if they: (1) employed an observational case-control design; (2) analyzed oral microbiota and reported diversity or abundance metrics; (3) included participants with clinically diagnosed mental disorders (any age) and matched healthy controls. Two authors (Dingxin Cao and Jun Yang) independently screened records, with discrepancies resolved through discussion or consultation with a third author (Yan Tu).

### Data extraction

Two authors (Dingxin Cao and Jun Yang) extracted data using a predefined template, cross-checked for consistency. Key extracted information included publication details, participant demographics, clinical characteristics, and methodological parameters. Primary outcomes focused on oral microbiota composition at the community level (alpha and beta diversity) and genus-level taxonomic findings (relative abundance). Alpha diversity reflects microbial richness (number of species) and evenness (distribution abundance) within individual samples, enabling cross-group comparisons to assess condition-specific effects. Beta diversity measures between-sample differences, evaluating overall community similarity. This analysis identified whether patient microbiomes diverged significantly from healthy controls (individuals without diagnosed mental disorders).

### Quality assessment

The 20 included studies were critically appraised using the Joanna Briggs Institute (JBI) checklist for case-control studies [15]. None were excluded due to methodological quality concerns. The detailed assessment is available in Table 1.

**Table 1.** JBI critical appraisal checklist for analytical cross sectional studies.

Study (First Author&Year)	1. Were the criteria for inclusion in the sample clearly defined?	2. Were the study subjects and the setting described in detail?	3. Was the exposure measured in a valid and reliable way?	4. Were objective, standard criteria used for measurement of the condition?	5. Were confounding factors identified?	6. Were strategies to deal with confounding factors stated?	7. Were the outcomes measured in a valid and reliable way?	8. Was appropriate statistical analysis used?
Cannon et al. [32]	yes	yes	yes	yes	yes	yes	yes	no
Evenepoel et al. [33]	yes	yes	yes	yes	yes	yes	yes	yes
Oda et al. [19]	yes	yes	yes	yes	yes	yes	yes	yes
Zhang et al. [20]	yes	no	yes	yes	yes	yes	yes	unclear
Abdulhaq et al. [21]	yes	yes	yes	yes	yes	yes	yes	unclear
Ragusa et al. [30]	yes	yes	yes	yes	yes	yes	yes	yes
Forsyth et al. [36]	yes	no	no	yes	yes	yes	yes	unclear
Kong et al. [22]	yes	yes	yes	yes	yes	yes	yes	yes
Hicks et al. [34]	yes	yes	yes	yes	yes	yes	yes	yes
Qiao et al. [23]	yes	yes	yes	yes	yes	yes	yes	yes
Lin et al. [37]	yes	yes	yes	yes	yes	yes	yes	unclear
Ling et al. [27]	yes	yes	yes	yes	yes	yes	yes	yes
Lee et al. [38]	yes	yes	yes	yes	yes	yes	yes	yes
Qing et al. [31]	yes	yes	yes	yes	yes	yes	yes	yes
Castro-Nallar et al. [25]	yes	yes	yes	yes	yes	yes	yes	yes
Lou et al. [26]	yes	yes	yes	yes	yes	yes	yes	unclear
Wingfield et al. [35]	yes	yes	yes	yes	yes	yes	yes	yes
Hu et al. [27]	yes	yes	yes	yes	yes	yes	yes	yes
Domènech et al. [28]	yes	yes	yes	yes	yes	yes	yes	yes
Xie et al. [29]	yes	yes	yes	yes	yes	yes	yes	yes

### Quantitative synthesis

For metrics reported in ≥ 10 studies, we performed meta-analyses of alpha diversity differences between case and control groups using random-effects models. Pooled effect estimates (with 95% confidence intervals) were visualized using forest plots. Effect sizes were interpreted using Cohen’s criteria: small (0.2), moderate (0.5), and large (0.8). Statistical heterogeneity was assessed via *I^2^* values: Negligible heterogeneity ( < 10%), Low heterogeneity (10–39%), Moderate heterogeneity (40–59%), High heterogeneity (60–89%), or Very high heterogeneity (≥90%) [16].

High heterogeneity (I^2^ ≥60%) was explored through preplanned subgroup analyses by diagnosis and geographic region (Eastern vs. Western studies). Publication bias was assessed via funnel plot symmetry using Egger’s test. All analyses were conducted in R v4.4.2 (meta and metafor packages; R Foundation) [17], with statistical significance set at *p* < 0.05.

### Qualitative synthesis

Given both the limited overlap in findings across studies reporting oral microbial relative abundance and the need for more stringent validation of genus-level differential abundance results to account for multiple comparisons, we conducted a qualitative synthesis. To minimize false positives, results reported by only one study or research group were excluded, as these might reflect method- or population-specific biases. To identify potentially condition-specific and shared alterations, we performed within-diagnosis and cross-diagnosis comparisons. First, taxa reported in at least two studies were categorized as increased, decreased, or inconsistent within a diagnosis. Inconsistency was defined as < 75% agreement across studies. Consistent findings in two studies were flagged for future validation; those in ≥ 3 studies (from ≥2 research groups) were considered robust associations. Taxa altered in only one disorder (with directional consistency) were classified as potentially condition-specific candidates [18]. Conversely, alterations recurring across disorders with overlapping symptoms or pathophysiology were deemed potentially transdiagnostic. Differences shared across unrelated diagnostic categories were interpreted as general disease responses.

## Results

### Search results

**Figure 1.**
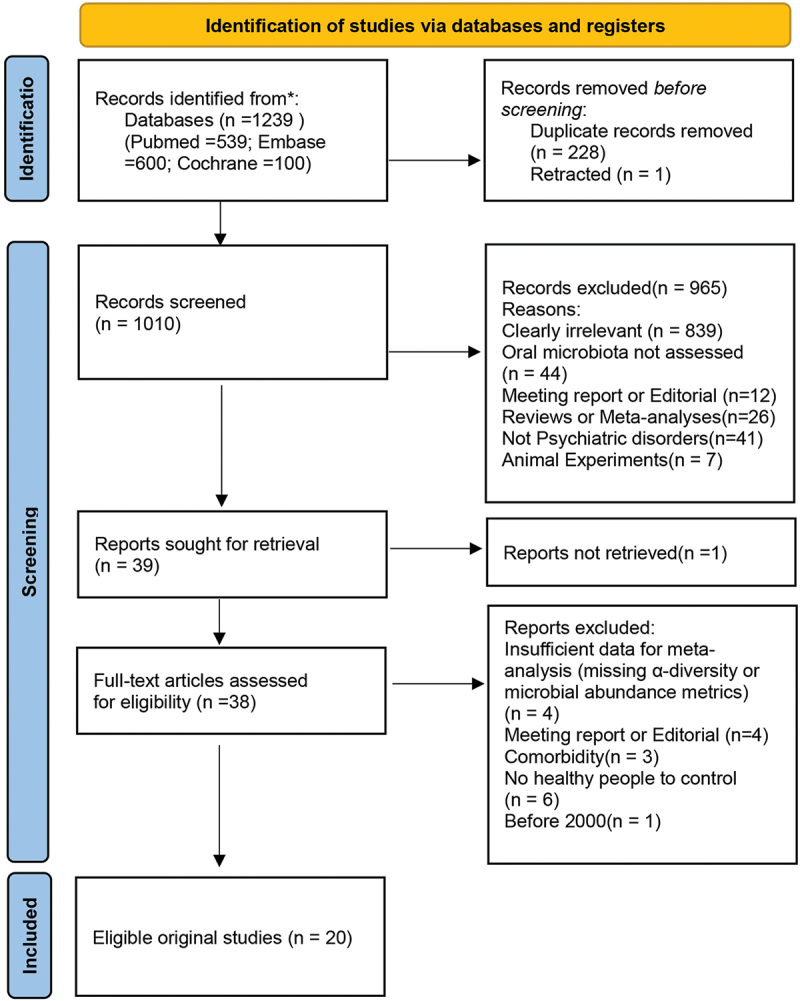
Presents the PRISMA flowchart of the study identification process.

Twenty studies met the inclusion criteria, covering six mental disorders: ASD, SZ, MDD, AUD, OCD, and PD. The most studied conditions were ASD, followed by SZ and MDD. The PRISMA flow diagram illustrating study selection is shown in Figure 1.

### Characteristics of included studies

Twenty case-control studies were included, comprising 1,034 patients and 840 controls (see Table 2). Most studies (13 [65%]) were conducted in Westernized populations (United States, Europe, Saudi Arabia; grouped by typical diet/lifestyle patterns), while 7 (35%) focused on East Asian populations (China). Studies were similar in exclusion criteria; however, only 5 of 10 adult studies (50%) reported participants’ smoking status, among which 4 implemented appropriate controls for smoking. Psychiatric medication use varied substantially: 1 study (5%) included drug-free or medication-resistant groups, 2 (10%) analyzed treated cohorts, and the remaining 17 (85%) did not report medication controls. Saliva sample processing methods also differed markedly. 16S rRNA sequencing was most common (17 [85%]), followed by metatranscriptomics (1 [5%]) and metagenomic sequencing (2 [10%]). Since the choice of reference databases can influence microbiome analysis results, we systematically documented the databases used across studies. Among the included studies, 6 (30%) failed to report their reference databases. The most frequently used databases were Greengenes and the Human Oral Microbiome Database (4 [20%]), followed by the Ribosomal Database Project (3 [15%]). Although all 20 studies were published after 2013, 7 of them (35%) still adopted the DSM-IV criteria, 1 (5%) did not report the diagnostic criteria used, and the remaining 12 studies (60%) employed the DSM-V criteria.

**Table 2.** Key sample and methodology characteristics of case-control comparisons of the oral microbiome by disorder.

Disorder	Study	Country	Sample size n	Mean Age(Year)	% Female	Mean BMI	% Smokers	% Patients on medication	Sequencing	Diversity assessments	reference databases	diagnostic criteria
ASD	Cannon et al. [32]	America	*p* = 30 HC = 30	*p* = 11 HC = 10	*p* = 6.7% HC = 40%	nr	*p* = 0% HC = 0%	nr	Metatranscriptomics	α: Shannon β: not measured	nr	nr
ASD	Evenepoel et al. [33]	Belgium	*p* = 80 HC = 40	*p* = 10.5 HC = 10.3	*p* = 20% HC = 20%	nr	*p* = 0% HC = 0%	nr	16S rRNA V3-V4	α: Observed sp., Chao 1, Pielou, Shannon, Simpson’s β: Bray-Curtis	Human Oral Microbiome Database	DSM-V
ASD	Oda et al. [18]	Turkey	*p* = 9 HC = 9	*p* = 3.7 HC = 3.9	*p* = 20% HC = 21%	nr	*p* = 0% HC = 0%	nr	16S rRNA V1-V9	α: ACE, Chao1, Shannon, Observed sp.,Simpson’s, InvSimpson, Fisher, β: UniFrac (weighted & unweighted)	Silva-132–99-nb	DSM-V
ASD	Zhang et al. [20]	China	*p* = 10 HC = 10	*p* = 4.4 HC = 3.9	nr	nr	*p* = 0% HC = 0%	nr	16S rRNA V4-V5	α: ACE, Chao1, Shannon, Simpson’s,Observed sp β: measured, nr	nr	DSM-V
ASD	Abdulhaq et al. [21]	Kingdom of Saudi Arabia	*p* = 25 HC = 38	*p* = 9.24 HC = 10.03	*p* = 36% HC = 52.6%	nr	*p* = 0% HC = 0%	nr	16S rRNA V1-V3	α: Observed sp., Chao 1, Shannon, Simpson’s β: measured, nr	nr	DSM-V
ASD	Ragusa et al. [30]	Italy	*p* = 76 HC = 39	*p* = 6.9 HC = 6.9	*p* = 21.1% HC = 28.2%	nr	*p* = 0% HC = 0%	nr	16S rRNA V3-V4	α: Chao1, Shannon β: UniFrac (weighted & unweighted)	Greengenes	DSM-V
ASD	Forsyth et al. [36]	America	*p* = 11 HC = 10	*p* = 10.68 HC = nr	*p* = 9.1% HC = 20%	nr	*p* = 0% HC = 0%	nr	16S rRNA V3-V4	α: Observed sp. β: measured, nr	Ribosomal Database Project、NT-16S	DSM-V
ASD	Kong et al. [22]	America	*p* = 19 HC = 20	*p* = 15 HC = 29	*p* = 25% HC = 58%	nr	*p* = 0% HC = 0%	nr	16S rRNA V3-V4	α: Shannon, Faith’s PD, Simpson’s β: UniFrac (weighted & unweighted), Bray-Curtis, Jaccard	Greengenes	DSM-V
ASD	Hicks et al. [34]	America	*p* = 180 HC = 106	*p* = 4.4 HC = 3.6	*p* = 14.4% HC = 39.6%		*p* = 0% HC = 0%	nr	Shotgun Metagenomics	α: Shannon β: Bray-Curtis	Human Microbiome Project	DSM-V
ASD	Qiao et al. [23]	China	*p* = 32 HC = 27	*p* = 10 HC = 10.2	*p* = 15.6% HC = 22.2%	*p* = 19.6 HC = 19.9	*p* = 0% HC = 0%	nr	16S rRNA V3-V4	α: ACE, Shannon, Shannoneven β: UniFrac (weighted & unweighted)	Human Oral Microbiome Database	DSM-V
SZ	Lin et al. [37]	America	*p* = 110 HC = 103	*p* = 37.67 HC = 38.42	*p* = 17.3% HC = 28.2%	nr	nr	*p* = 100% HC = 0%	16S rRNA V4	α: not measured β: UniFrac (weighted), Bray-Curtis	Greengenes	DSM-IV
SZ	Ling et al. [24]	China	*p* = 118 HC = 97	*p* = 75.18 HC = 74.68	*p* = 45.8% HC = 49.5%	*p* = 23.6 HC = 24.59	*p* = 12.7% HC = 12.4%	nr	16S rRNA V3-V4	α: Shannon, Simpson’s, Inv. Simpson, ACE, Chao1, Observed sp. β: UniFrac (weighted & unweighted), Bray-Curtis, Jaccard	nr	DSM-IV
SZ	Lee et al. [38]	America	*p* = 9 HC = 8	*p* = 39.1 HC = 36.9	*p* = 66.7% HC = 62.5%	*p* = 29.2 HC = 26.6	nr	*p* = 86.7% HC = 0%	16S rRNA V4	α: observed features β: UniFrac (unweighted)	nr	DSM-V
SZ	Qing et al. [31]	China	*p* = 85 HC = 80	*p* = 25.66 HC = 22.88	*p* = 42.4% HC = 48.8%	nr	*p* = 0% HC = 5%	*p* = 0% HC = 0%	16S rRNA V4	α: ACE, Shannon, Chao1 β: UniFrac (weighted & unweighted)	Ribosomal Database Project	DSM-IV
SZ	Castro-Nallar et al. [25]	America	*p* = 16 HC = 16	*p* = 34.7 HC = 34.3	*p* = 43.8% HC = 43.8%	*p* = 34.7 HC = 25.5	*p* = 62.5% HC = 0%	nr	shotgun metagenomics	α: ACE, Shannon, Chao1, Simpson’s, InvSimpson, Fisher, Observed sp. β: Bray-Curtis	Human Microbiome Project	DSM-IV
MDD	Lou et al. [26]	China	*p* = 87 HC = 70	*p* = 48.77 HC = 41.53	nr	*p* = 23.53 HC = 23.56	nr	nr	16S rRNA	α: ACE, Shannon, Chao1, Observed sp. β: measured, nr	nr	DSM-V
MDD	Wingfield et al. [35]	England	*p* = 40 HC = 43	*p* = 21.8 HC = 20.4	*p* = 72.5% HC = 69.8%	nr	*p* = 45% HC = 20.9%	nr	16S rRNA V3-V4	α: Shannon, Observed sp., Inv. Simpson β: Bray-Curtis	Human Oral Microbiome Database	DSM-IV
AUD	Hu et al. [27]	China	*p* = 33 HC = 21	*p* = 44 HC = 43	*p* = 0% HC = 0%	*p* = 21.60 HC = 22.87	*p* = 90.9% HC = 76.2%	nr	16S rRNA V3-V4	α: ACE, Shannon, Chao1, Observed sp., Coverage, Simpson’s β: UniFrac (weighted)	Greengenes	DSM-IV
OCD	Domènech et al. [28]	Spain	*p* = 38 HC = 33	*p* = 40.16 HC = 36	*p* = 52.6% HC = 54.5%	nr	nr	nr	16S rRNA V3-V4	α: ACE, Shannon, Chao1, Simpson’s, Faith’s PD, Observed sp. β: UniFrac (weighted & unweighted), Bray-Curtis, Canberra, Jensen-Shannon	SILVA	DSM-IV
PD	Xie et al. [29]	China	*p* = 26 HC = 40	*p* = 41.4 HC = 39.6	*p* = 46.2% HC = 45.0%	*p* = 24.1HC = 22.6	nr	nr	16S rRNA V3-V4	α: Shannon, Chao1, Simpson’s, Observed sp. β: UniFrac (weighted)	Ribosomal Database Project	DSM-V

### Alpha diversity

Nineteen of the 20 studies reported alpha diversity metrics. Ten indices were used to assess alpha diversity, including estimates of richness (Observed Species, Chao1, ACE), evenness (Pielou, Shannon Even), diversity (Shannon, Simpson, inverse Simpson, Fisher), and phylogenetic diversity (Faith’s PD). The most frequently applied indices were Observed Species, Chao1, Shannon, and Simpson.

**Figure 2.**
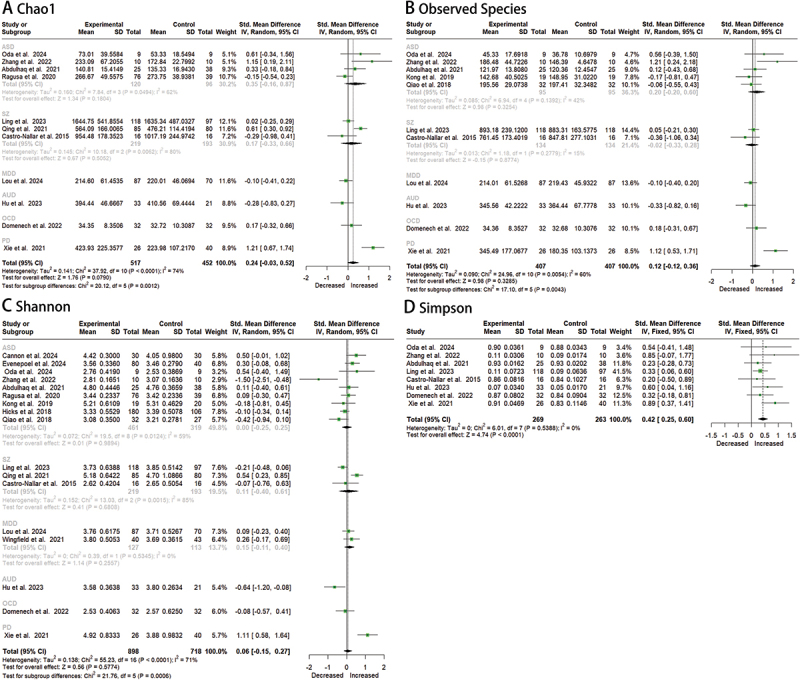
Forest plots of oral microbiota alpha-diversity in patients with mental disorders versus healthy controls.

Eleven studies (patients: *n* = 407; controls: *n* = 380) reported Observed Species data [19–29,23,25,27,30,31,33–35]. The pooled analysis revealed no significant difference between patients and controls (SMD = 0.12; 95% CI: −0.12 to 0.36; *p* = 0.0054), with substantial heterogeneity (*I*^2^ = 65%, *H* = 1.58) (Figure 2A). Subgroup analyses demonstrated a significant increase in Observed Species specifically in PD (SMD = 1.12; 95% CI: 0.53–1.71; *p* < 0.0001).

Eleven studies (patients: *n* = 517; controls: *n* = 452) provided Chao1 index data [19–21,24–31,33–35]. Under the random-effects model, no significant differences were observed (SMD = 0.25; 95% CI: −0.04 to 0.54; *p* = 0.0932; *I*^2^ = 74%) (Figure 2B). Notably, the fixed-effects model yielded a statistically significant effect (SMD = 0.18; 95% CI: 0.06–0.31; *p* = 0.0051), indicating that heterogeneity substantially influenced the results (*H* = 1.95, *I*^2^ = 74%). Subgroup analysis identified a marked elevation in PD (SMD = 1.21; 95% CI: 0.67–1.74; *p* < 0.0001). Given the high heterogeneity, random-effects model results were prioritized.

Seventeen studies (patients: *n* = 898; controls: *n* = 718) assessed Shannon diversity [19–35]. The pooled estimate showed no significant overall difference (SMD = 0.07; 95% CI: −0.03 to 0.17; *p* = 0.1839) (Figure 2C), with high heterogeneity (*I*^2^ = 71%, *H* = 1.86). Diagnostic subgroups exhibited divergent patterns: a significant decrease in AUD (SMD = −1.50; 95% CI: −2.52 to − 0.48) contrasted with an increase in PD (SMD = 1.11; 95% CI: 0.58–1.64).

Eight studies (patients: *n* = 269; controls: *n* = 263) evaluated Simpson Index, including 3 ASD, 2 SZ, 1 AUD, 1 OCD and 1 PD studies [19–21,24,25,27–29]. A statistically significant increase in diversity was observed in patients (SMD = 0.42; 95% CI: 0.25–0.60; *p* < 0.00001) (Figure 2D). Heterogeneity analysis demonstrated minimal between-study variation (tau^2^ = 0; *I*^2^ = 0%; *H* = 1.00), supported by a non-significant Q-test (*p* = 0.5390).

### Heterogeneity exploration (subgroup analysis and meta-regression)

To investigate sources of heterogeneity, subgroup analyses and meta-regressions were performed for metrics with sufficient studies and moderate-to-high heterogeneity (Observed Species, Chao1, Shannon).

Subgroup analyses by diagnosis revealed that the specific mental disorder (ASD, SZ, MDD, AUD, OCD, PD) significantly contributed to heterogeneity in alpha diversity. Between-subgroup heterogeneity tests showed substantial variation for Observed Species (Q = 17.10, df = 5, *p* = 0.0043), Chao1 (Q = 20.12, df = 5, *p* = 0.0012), and Shannon Index (Q = 21.74, df = 5, *p* = 0.0006). Notably, high residual heterogeneity persisted within ASD (*I^2^* = 62%) and SZ (*I^2^* = 80%) subgroups, suggesting additional unexplained variance from study design or sample characteristics. The PD subgroup demonstrated markedly larger effect sizes than other diagnoses, indicating distinct microbial alterations.

Subgroup analysis by geographic region (Eastern vs. Western studies) showed no significant association with outcomes (*p* > 0.05for all indices). Meta-regression analyses using study year and sequencing method as covariates revealed no significant moderating effects on effect sizes (regression coefficients *p* > 0.15). These findings indicate that moderate heterogeneity in Observed Species, Chao1, and Shannon results primarily stems from cross-diagnostic differences rather than geographic, temporal, or methodological factors.

### Sensitivity analysis

**Figure 3.**
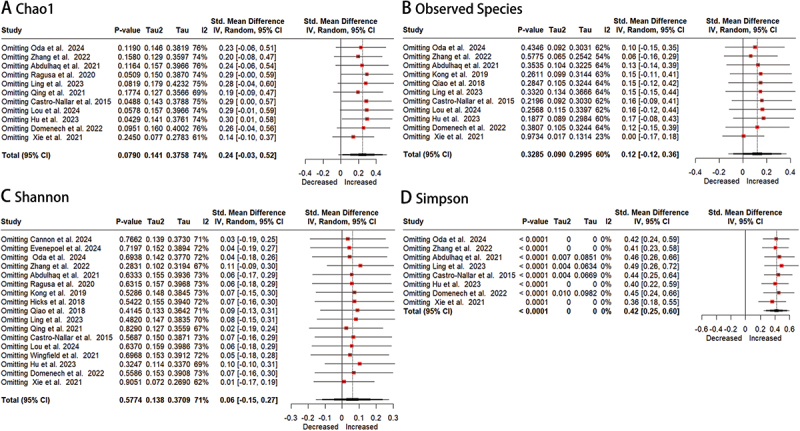
Sensitivity analysis forest plots of oral microbiota alpha-diversity in patients with mental disorders versus healthy controls.

A sensitivity analysis was conducted by sequentially excluding individual studies using a random-effects model to assess their impact on the overall findings. While the pooled standardized mean difference (SMD) and corresponding 95% confidence intervals (CI) exhibited some variability upon removal of specific studies, the central trend remained consistent across all iterations (Figure 3). This pattern demonstrates reasonable robustness of the primary outcomes.

### Publication bias analysis

Publication bias was assessed using Egger’s linear regression method to evaluate funnel plot asymmetry for the Observed Species, Chao1, and Shannon indices. No significant evidence of publication bias was detected across these measures.

**Figure 4.**
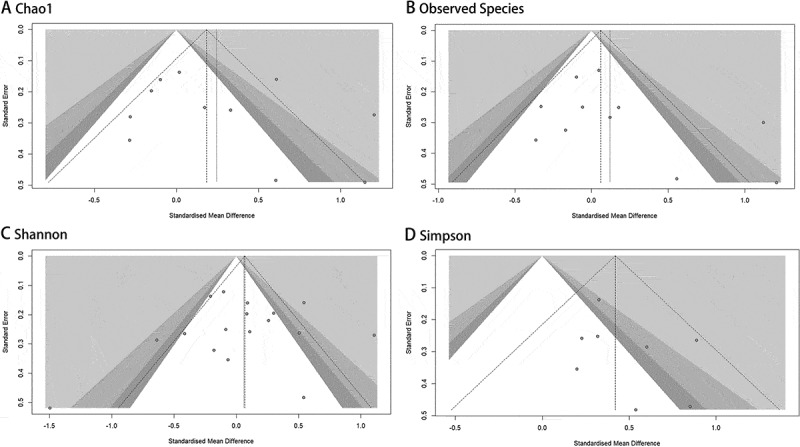
Funnel plots of oral microbiota alpha-diversity in patients with mental disorders versus healthy controls.

For the Simpson index, Egger’s test could not be performed due to the limited number of studies (*n* = 8), which falls below the recommended threshold of 10. Visual inspection of the contour-enhanced funnel plot revealed symmetrical data point distribution, with most studies demonstrating statistical significance (Figure 4). Notably, studies without statistical significance maintained symmetrical distribution in non-significant regions, collectively suggesting minimal likelihood of publication bias.

### Beta diversity

Comparisons of beta diversity between patient groups and controls were reported in 18 studies using multiple analytical measures (see Table 3) [19,21–31,33–38]. Ten studies demonstrated consistent statistically significant differences, seven showed consistent non-significant results, and one reported conflicting outcomes across different metrics. Condition-specific patterns emerged: Among ASD studies (*n* = 9), six revealed no significant clustering differences between patients and controls; In SZ research (*n* = 5), four studies identified significant dissimilarities; Both MDD studies (*n* = 2) reported significant variations; Single studies in AUD and PD similarly demonstrated significant differences; For OCD, while four measures showed non-significant results in the single available study, the Canberra metric indicated statistical significance. These findings demonstrate significant microbial composition differences in SZ and MDD patients compared with controls, indicating distinct microbial signatures in these conditions. In contrast, the inconsistent ASD findings may stem from methodological variations or clinical heterogeneity across ASD subtypes.

**Table 3.** Methodology and findings of the included studies assessing beta diversity for the patient vs. control group comparison.

Disorder	Study	Year	Metric	Analysis	Finding
ASD	Evenepoel et al.	2024	Bray-Curtis	ANOSIM	no sig. difference
ASD	Oda et al.	2024	weighted UniFrac unweighted UniFrac	PCoA, PERMANOVA	no sig. difference no sig. difference
ASD	Abdulhaq et al.	2021	–	–	no sig. difference
ASD	Ragusa et al.	2020	weighted UniFrac unweighted UniFrac	PCoA	no sig. difference no sig. difference
ASD	Forsyth et al.	2020	–	PCoA	no sig. difference
ASD	Kong et al.	2019	weighted UniFrac unweighted UniFrac Bray-CurtisJaccard	PERMANOVA	no sig. difference no sig. difference no sig. difference no sig. difference
ASD	Hicks et al.	2018	Bray-Curtis	PLS-DA	sig. different
ASD	Qiao et al.	2018	weighted UniFrac unweighted UniFrac	PCoA, PERMANOVA	sig. different sig. different
SZ	Lin et al.	2024	weighted UniFrac unweighted UniFrac Bray-Curtis	PCoA	sig. different sig. different sig. different
SZ	Ling et al.	2023	weighted UniFrac unweighted UniFrac Bray-CurtisJaccard	PCoA	sig. different sig. different sig. different sig. different
SZ	Lee et al.	2023	unweighted UniFrac	PERMANOVA	sig. different
SZ	Qing et al.	2021	weighted UniFrac unweighted UniFrac	PCoA	sig. different sig. different
SZ	Castro-Nallar et al.	2015	Bray-Curtis	–	no sig. difference
MDD	Lou et al.	2024	–	PCoA	sig. different
MDD	Wingfield et al.	2021	Bray-Curtis	PCoA	sig. different
AUD	Hu et al.	2023	weighted UniFrac	PCoA	sig. different
OCD	Domènech et al.	2022	weighted UniFrac unweighted UniFrac Bray-CurtisJensen-ShannonCanberra	PERMANOVA	no sig. difference no sig. difference no sig. difference no sig. difference sig. different
PD	Xie et al.	2021	weighted UniFrac	ANOSIM, PCoA	sig. different

### Differentially abundant microbial taxa

**Figure 5.**
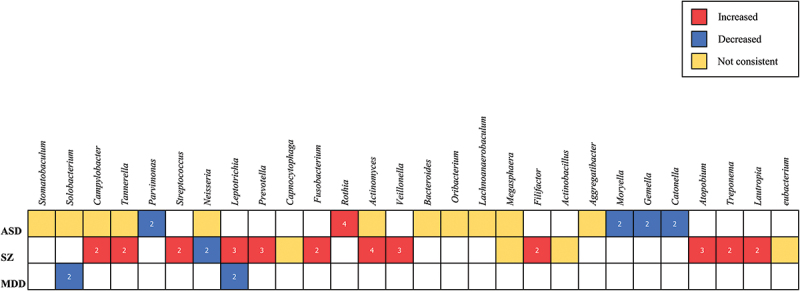
Differences in relative abundance of microbial genera reported by at least 2 studies within a diagnostic category.

The summarized comparisons within and across disorders with sufficient studies (ASD, SZ, and MDD) are presented in the figure 5 [19–26,30–38]. Empty cells denote cases that were ‘not examined’, ‘not reported’, or ‘not replicated’, while numerical values within cells indicate the number of studies supporting a given trend. High inconsistency was observed in microbial variations within individual disease categories, with most consensus findings being replicated by only 2 studies, necessitating further investigation. Microbial alterations demonstrating reproducibility across multiple studies were comparatively limited.

### Disease specificity

Limited evidence suggests condition-specific microbial patterns: Enrichment of *Rothia* and depletion of *Parvimonas*, *Moryella*, *Gemella*, and *Catonella* were observed in ASD. However, among 10 ASD studies, most findings were replicated in only two investigations, with *Rothia* enrichment being the most consistent observation (4/10 studies).

In SZ, enrichment was noted for *Campylobacter*, *Tannerella*, *Streptococcus*, *Leptotrichia*, *Prevotella*, *Fusobacterium*, *Actinomyces*, *Veillonella*, *Filifactor*, *Atopobium*, *Treponema*, and *Lautropia*, alongside *Neisseria* depletion. Similarly, most taxonomic alterations showed replication in two of five SZ studies, though *Leptotrichia*, *Prevotella*, *Actinomyces*, *Veillonella*, and *Atopobium* enrichment demonstrated stronger support (3–4 studies).

Both MDD studies consistently reported reduced *Solobacterium* and *Leptotrichia* abundance.

### Cross-diagnostic alterations

As shown in the Figure 5, our analysis identified no consistent microbial alterations shared across different diagnostic categories.

## Discussion

### Interpretation of α-diversity findings with heterogeneity analysis and β-diversity patterns

The current analysis revealed no significant differences in oral microbial α-diversity indices (including Chao1, Observed Species and Shannon) between mental disorder patients and healthy controls. The Observed Species index, which directly counts the number of distinct microbial taxa detected, indicates that the studied disorders did not significantly affect oral microbial richness. Similarly, the Chao1 index results, which account for potential undersampling through statistical estimation, showed no significant richness differences between patients with mental disorders and healthy controls after coverage adjustment. The Shannon index, incorporating both microbial richness and evenness in its calculation, further supported these findings, suggesting relative structural stability of oral microbiota in these clinical populations [39]. Notable exceptions emerged for specific diagnoses: PD patients exhibited significantly elevated α-diversity, while AUD patients demonstrated reduced Shannon indices. These patterns may indicate condition-specific microbial community dynamics.

The seminal study by Xie et al. first identified significant oral microbiome alterations in Chinese PD patients compared to healthy controls [29]. Their findings demonstrated elevated α-diversity across most metrics (Observed Species, Chao1, and Shannon indices), while the increased Simpson index paradoxically suggests reduced diversity due to its emphasis on dominant species probability [40]. his apparent contradiction reflects the distinct ecological information captured by different indices: the Simpson index specifically indicates an increased proportion of dominant species in PD patients’ oral microbiota, potentially reflecting environmental selection pressures or ecological imbalance. This high-diversity microbiome profile pattern has been associated with poorer oral health status and may facilitate chronic oral inflammation [41]. Supporting this mechanism, PD patients show increased serum levels of inflammatory mediators including IL-6 and IL-1β [42]. Persistent inflammatory and immune responses could compromise blood-brain barrier integrity, potentially enabling bacterial translocation to neural tissues and subsequent neurological impact. This may establish a bidirectional relationship where anxiety symptoms exacerbate microbial alterations through hypothalamic-pituitary-adrenal (HPA) axis activation. PD-associated anxiety and stress states stimulate cortisol secretion [43,44], which modifies oral environments (e.g. salivary composition) to favor growth of specific taxa like *Fusobacteria* while suppressing others [45]. These documented mechanisms provide a plausible framework for understanding our findings, though direct causal relationships between oral microbiome changes and neurological symptoms require further investigation.

Hu et al. demonstrated reduced alpha-diversity in both oral(Shannon index) and gut microbiomes(Sob index, Chao1 index, ACE index, and Coverage index) of AUD patients [27], suggesting alcohol-induced oral-gut microbiome disruption [46]. However, these findings contradict Liao et al.‘s observations in general alcohol-consuming populations [47]. While small sample effects and methodological differences between studies may partially explain this discrepancy, it also suggests that AUD-specific microbial alterations extend beyond alcohol consumption itself, providing partial evidence for an oral microbiome-brain axis [48].

The elevated Simpson indices in patients with mental disorders suggest decreased microbial evenness with dominant species predominance, particularly evident in SZ and ASD [39]. This is further supported by Ling et al.‘s study, which showed that the genus Streptococcus accounted for approximately 33% of the oral microbiota in SZ patients, compared to only 26% in healthy controls [24]. Notably, while alpha diversity metrics (e.g. Shannon, Chao1) provide a useful overview of microbial community richness and evenness, their biological interpretation remains limited. These indices summarize community structure but do not directly reflect functional differences, host-microbe interactions, or ecological dynamics underlying the observed differences.

Subgroup analyses revealed that heterogeneity primarily stemmed from inter-diagnostic differences rather than geographic, temporal, or sequencing methodology variables, supporting condition-specific microbial alterations. However, residual heterogeneity within subgroups highlights limitations from insufficient sample sizes and potential confounding by factors including smoking status and psychotropic medication use, underscoring the need for standardized methodologies in future research.

Beta-diversity analysis revealed significant microbial composition differences in SZ and MDD patients versus controls, suggesting more distinct microbial signatures in these conditions. The inconsistent ASD findings (6/9 studies showing non-significant differences) may stem from methodological variations (e.g. sample sources, sequencing depth) or clinical heterogeneity across ASD subtypes. The more permissive threshold for reporting beta diversity results (compared to our stringent criteria for differential abundance findings) reflects the complementary value of both community-level and taxon-specific analyses – where whole-ecosystem approaches may detect subtle but biologically important shifts even when individual taxa variations fall below strict significance thresholds.

### Condition-specific microbial alterations

In ASD, enrichment of *Rothia* and depletion of *Parvimonas*, *Moryella*, *Gemella*, and *Catonella* were observed. However, most findings were replicated in only a limited number of studies, with *Rothia* enrichment showing relatively stronger validation across investigations. No direct evidence currently links *Rothia* to ASD pathogenesis. As the third most abundant genus in human breast milk (HBM) microbiota [49], *Rothia* normally contributes to oral and gut health as part of the commensal microbiome. The observed *Rothia* dominance in ASD oral microbiota aligns with elevated Simpson indices from meta-analyses, reflecting reduced microbial evenness and predominant species overgrowth. *Rothia* – a Gram-positive cocci genus extensively colonizing the oral cavity – has been associated with severe infections in immunocompromised hosts [50]. This genus may modulate host immunity by stimulating pro-inflammatory cytokine production (e.g. TNF-α and IL-6) in oral and intestinal environments [51].

In SZ, enrichment of *Leptotrichia*, *Prevotella*, *Actinomyces*, *Veillonella*, and *Atopobium* was observed. Notably, these bacteria are all H_2_S-producing species [52]. The increase of such H2S-producing bacteria in the oral cavity may compromise local tissues, such as gums and mucosa, thereby triggering periodontitis, a condition closely related to the inflammatory state in schizophrenia [53]. Meanwhile, the H2S produced by these bacteria can induce low-grade systemic inflammation, leading to the release of pro-inflammatory cytokines (e.g. TNF-α, IL-6), which in turn affect brain function, disrupt the blood-brain barrier (BBB), and potentially lead to the manifestation of cognitive impairments and psychotic symptoms. Furthermore, H2S may also enter the brain via the bloodstream or neural pathways (such as the trigeminal nerve), influencing neural function. Studies have found that H2S levels are correlated with peripheral pro-inflammatory cytokines (e.g. IL-6, S100B), suggesting its potential role in systemic inflammation [31]. Although Endogenously produced hydrogen sulfide (H_2_S), particularly by gut microbiota, plays crucial physiological roles including vasodilation, neuroprotection through oxidative stress reduction, and modulation of innate/adaptive immune responses [54], However, its overproduction, as demonstrated by Ide et al. in the pathophysiology of schizophrenia, may disrupt redox balance and immune regulation [55]. This biphasic nature (i.e. protective and pathological) of microbially produced H2S is particularly noteworthy in the context of schizophrenia-associated alterations in the oral microbiome.

In MDD, both studies reported reduced abundance of *Solobacterium* and *Leptotrichia*. Lou et al. developed a co-occurrence network through Spearman correlation analysis to explore interactions among oral bacteria, salivary metabolites, and depression severity [26]. This network revealed strong associations between *Leptotrichia*, lipid-related salivary metabolites, and specific organic acids/derivatives, which suggests *Leptotrichia* may participate in depression-related metabolic pathways. However, the evidence linking *Leptotrichia* to depression remains preliminary, and further research is needed to validate these associations and elucidate their potential mechanisms.

### Implications of cross-diagnostic commonality deficits

The observed lack of overlapping oral microbial alterations across mental disorders contrasts with established cross-diagnostic patterns in gut microbiome research (characterized by reduced *Enterococcus* and *Bifidobacterium* alongside enriched *Eggerthella*) [18]. This divergence may suggest greater condition-specificity in oral microbial contributions to psychopathology, or stronger regulation by local microenvironmental factors such as salivary pH levels and oral hygiene practices, or functional redundancy among phylogenetically distinct taxa – where different microbial species may fulfill similar metabolic roles, making shared pathophysiology manifest downstream (e.g. in microbial metabolites or host immune responses) rather than through conserved taxonomic shifts [56]. Notably, current cross-diagnostic interpretations remain constrained by limited data availability for AUD, OCD, and PD – each represented by only one study. Expanded investigations targeting these conditions are required to validate the generalizability of these findings and elucidate the complex mechanistic roles of oral microbiota in psychiatric pathophysiology.

### Limitations and future directions

#### Key limitations

Current research demonstrates insufficient control of critical confounding variables. Among the included adult studies, only 50% accounted for smoking status, while most failed to adequately address the impact of psychotropic medications (e.g. antidepressants, antipsychotics) on microbial profiles – factors likely introducing substantial bias.

Lenartova et al. have demonstrated significant associations between oral microbiota and periodontal health status [57]. However, none of the 20 studies included in our analysis investigated the periodontal conditions of participants. This lack of data may interfere with our experimental results and represents a potential confounding factor. Future studies should systematically address this limitation through clinical oral examinations and standardized periodontal assessments.

Significant variations exist across studies in sample collection (saliva vs. tongue coating), sequencing platforms (16S rRNA vs. metagenomics), and analytical pipelines, limiting result comparability and conclusion generalizability.

The field has neglected critical clinical dimensions including disease subtypes (e.g. first-episode vs. chronic schizophrenia) and illness progression stages, potentially obscuring essential microbial dynamics.

Most studies remain observational, with limited mechanistic exploration. Future work should integrate animal models and in vitro approaches to establish causal relationships and elucidate specific pathways linking oral microbiota with neurological functions.

The small number of qualifying studies in this emerging field (*n* = 20) may reduce statistical power, increase susceptibility to publication bias, and limit the robustness of meta-analytic conclusions.

Unlike psychiatric conditions such as MDD or SZ, ASD is classified as a neurodevelopmental disorder. This categorical distinction may influence mechanistic interpretations. Future research should therefore explore transdiagnostic frameworks that explicitly incorporate such diagnostic differences.

#### Future directions

Priority should be given to multi-center, large-sample cohort studies implementing standardized protocols for sample collection, sequencing, and data analysis. As advocated and implemented by Malan-Müller et al., rigorous control of confounding factors will enhance result reliability and cross-study comparability [58].

Lin et al. discussed recent technological innovations in characterizing the oral microbiome using omics approaches, including high-throughput sequencing, long-read platforms, single-cell genomics, and advanced imaging modalities ，which highlights the potential of these technologies to provide comprehensive taxonomic and functional profiling [59]. Combining metagenomic, metabolomic, and host immune profiling data will enable comprehensive characterization of oral microbiota’s functional roles in psychopathology. This integrated approach will provide scientific foundations for targeted therapeutic interventions.

Systematic investigation of oral microbiota as non-invasive biomarkers is warranted. The development of saliva-based rapid detection technologies could offer novel approaches for early diagnosis and personalized treatment strategies in mental disorders [60].

## Conclusion

This systematic review and meta-analysis reveals distinct microbial signatures in the oral microbiome of individuals with mental disorders. Focusing on six major disorders – ASD, SZ, MDD, AUD, OCD, and PD – we identified significant alterations in microbial diversity metrics. Elevated Simpson indices and marked beta-diversity variations in SZ/MDD patients provide critical diagnostic clues. Our work establishes the first comprehensive characterization of condition-specific oral microbial patterns: *Rothia* enrichment in ASD, marked proliferation of H2S-producing genera (*Leptotrichia*, *Prevotella*, *Actinomyces*, *Veillonella*, *Atopobium*) in SZ, and reduced *Solobacterium*/*Leptotrichia* abundance in MDD. These findings suggest that the oral microbiome may be a candidate factor associated with these mental disorders. Further investigation is warranted to explore the potential diagnostic applications and microbial-targeted therapeutic strategies.

## Data Availability

Data sharing is not applicable to this article as no new data were created or analyzed in this study.

## Other information registration

The protocol for this systematic review was prospectively registered with PROSPERO (Registration ID: CRD42024645555).

## References

[1] Hossain MM, Khan N, Sultana A, et al. Prevalence of comorbid psychiatric disorders among people with autism spectrum disorder: an umbrella review of systematic reviews and meta-analyses. Psychiatry Res. 2020;287:112922. doi: 10.1016/j.psychres.2020.112922 32203749

[2] Kessler RC, Gruber M, Hettema JM, et al. Comorbid major depression and generalized anxiety disorders in the National comorbidity survey follow-up. Psychol Med. 2008;38(3):365–19. doi: 10.1017/S0033291707002012 18047766 PMC2745899

[3] Jauhar S, Johnstone M, McKenna PJ. Schizophrenia. Lancet. 2022;399(10323):473–486. doi: 10.1016/S0140-6736(21)01730-X 35093231

[4] Arias D, Saxena S, Verguet S. Quantifying the global burden of mental disorders and their economic value. eClinicalmedicine. 2022;54:101675. doi: 10.1016/j.eclinm.2022.101675 36193171 PMC9526145

[5] Castaldelli-Maia JM, Bhugra D. Analysis of global prevalence of mental and substance use disorders within countries: focus on sociodemographic characteristics and income levels. Int Rev Psychiatry. 2022;34(1):6–15. doi: 10.1080/09540261.2022.2040450 35584016

[6] Bonnechère B, Amin N, van Duijn C. The role of gut microbiota in neuropsychiatric diseases – creation of an atlas-based on quantified evidence. Front Cell Infect Microbiol. 2022;12. doi: 10.3389/fcimb.2022.831666 PMC8964285 35360098

[7] Cryan JF, O’Riordan KJ, Cowan CSM, et al. The microbiota-gut-brain axis. Physiol Rev. 2019;99(4):1877–2013. doi: 10.1152/physrev.00018.2018 31460832

[8] Torres-Fuentes C, Schellekens H, Dinan TG, et al. The microbiota–gut–brain axis in obesity. Lancet Gastroenterol Hepatol. 2017;2:747–756. doi: 10.1016/S2468-1253(17)30147-4 28844808

[9] Góralczyk-Bińkowska A, Szmajda-Krygier D, Kozłowska E. The microbiota–gut–brain axis in psychiatric disorders. Int J Mol Sci. 2022;23(19):11245. doi: 10.3390/ijms231911245 36232548 PMC9570195

[10] Li Z, Liu S, Liu F, et al. Gut microbiota and autism spectrum disorders: a bidirectional Mendelian randomization study. Front Cell Infect Microbiol. 2023;13:1267721. doi: 10.3389/fcimb.2023.1267721 38156319 PMC10753022

[11] Minty M, Canceil T, Serino M, et al. Oral microbiota-induced periodontitis: a new risk factor of metabolic diseases. Rev Endocr Metab Disord. 2019;20(4):449–459. doi: 10.1007/s11154-019-09526-8 31741266

[12] Malamud D. Saliva as a diagnostic fluid. Dent Clin North Am. 2011;55(1):159–178. doi: 10.1016/j.cden.2010.08.004 21094724 PMC3011946

[13] Hutton B, Salanti G, Caldwell DM, et al. The PRISMA extension statement for reporting of systematic reviews incorporating network meta-analyses of health care interventions: checklist and explanations. Ann Intern Med. 2015;162(11):777–784. doi: 10.7326/M14-2385 26030634

[14] Cochrane handbook for systematic reviews of interventions. Available from: https://training.cochrane.org/handbook/current

[15] Chapter 7: systematic reviews of etiology and risk | semantic scholar. Available from: https://www.semanticscholar.org/paper/Chapter-7%3A-Systematic-Reviews-of-Etiology-and-Risk-Moola-Munn/9d00e4cbc9d6f99b33a731358bc04a35d0ab4c48

[16] Schober P, Mascha EJ, Vetter TR. Statistics from a (agreement) to Z (z score): a guide to interpreting common measures of association, agreement, diagnostic accuracy, effect size, heterogeneity, and reliability in medical research. Anesth & Analg. 2021;133(6):1633. doi: 10.1213/ANE.0000000000005773 34633993

[17] Balduzzi S, Rücker G, Schwarzer G. How to perform a meta-analysis with R: a practical tutorial. Evid Based Ment Health. 2019;22(4):153–160. doi: 10.1136/ebmental-2019-300117 31563865 PMC10231495

[18] Nikolova VL, Smith MRB, Hall LJ, et al. Perturbations in gut microbiota composition in psychiatric disorders. JAMA Psychiatry. 2021;78(12):1–12. doi: 10.1001/jamapsychiatry.2021.2573 PMC8444066 34524405

[19] Oda G, Kaya DE, Kaynar TB, et al. Comparison of the oral microbiota of children with autism spectrum disorder in primary dentition with neurotypical controls. Res In Autism Spectr Disord. 2024;118:102480. doi: 10.1016/j.rasd.2024.102480

[20] Zhang Y, Zhang X. A study on bacteria in saliva of autistic children at early life. Jundishapur J Microbiol. 2022;15(4). doi: 10.5812/jjm-123331

[21] Abdulhaq A, Halboub E, Homeida HE, et al. Tongue microbiome in children with autism spectrum disorder. J Oral Microbiol. 2021;13(1):1936434. doi: 10.1080/20002297.2021.1936434 34211637 PMC8221129

[22] Kong X, Liu J, Cetinbas M, et al. New and preliminary evidence on altered oral and gut microbiota in individuals with autism spectrum disorder (ASD): implications for ASD diagnosis and subtyping based on microbial biomarkers. Nutrients. 2019;11(9):2128. doi: 10.3390/nu11092128 31489949 PMC6770733

[23] Qiao Y, Wu M, Feng Y, et al. Alterations of oral microbiota distinguish children with autism spectrum disorders from healthy controls. Sci Rep. 2018;8(1):1597. doi: 10.1038/s41598-018-19982-y 29371629 PMC5785483

[24] Ling Z, Cheng Y, Liu X, et al. Altered oral microbiota and immune dysfunction in Chinese elderly patients with schizophrenia: a cross-sectional study. Transl Psychiatry. 2023;13(1):383. doi: 10.1038/s41398-023-02682-1 38071192 PMC10710460

[25] Castro-Nallar E, Bendall ML, Pérez-Losada M, et al. Composition, taxonomy and functional diversity of the oropharynx microbiome in individuals with schizophrenia and controls. Peer J. 2015;3:e1140. doi: 10.7717/peerj.1140 26336637 PMC4556144

[26] Lou F, Luo S, Kang N, et al. Oral microbiota dysbiosis alters chronic restraint stress-induced depression-like behaviors by modulating host metabolism. Pharmacol Res. 2024;204:107214. doi: 10.1016/j.phrs.2024.107214 38763328

[27] Hu L, Ni Z, Zhao K, et al. The association between oral and gut microbiota in male patients with alcohol dependence. Front Microbiol. 2023;14:1203678. doi: 10.3389/fmicb.2023.1203678 37577447 PMC10422022

[28] Domènech L, Willis J, Alemany-Navarro M, et al. Changes in the stool and oropharyngeal microbiome in obsessive-compulsive disorder. Sci Rep. 2022;12(1):1448. doi: 10.1038/s41598-022-05480-9 35087123 PMC8795436

[29] Xie Z, Jiang W, Deng M, et al. Alterations of oral microbiota in patients with panic disorder. Bioengineered. 2021;12(1):9103–9112. doi: 10.1080/21655979.2021.1994738 34666612 PMC8806997

[30] Ragusa M, Santagati M, Mirabella F, et al. Potential associations among alteration of salivary miRnas, saliva microbiome structure, and cognitive impairments in autistic children. Int J Mol Sci. 2020;21(17):6203–6224. doi: 10.3390/ijms21176203 32867322 PMC7504581

[31] Qing Y, Xu L, Cui G, et al. Salivary microbiome profiling reveals a dysbiotic schizophrenia-associated microbiota. NPJ Schizophr. 2021;7(1):51. doi: 10.1038/s41537-021-00180-1 34711862 PMC8553823

[32] Cannon M, Toma R, Ganeshan S, et al. Salivary transcriptome and mitochondrial analysis of autism spectrum disorder children compared to healthy controls. NeuroSci. 2024;5(3):276–290. doi: 10.3390/neurosci5030022 39483288 PMC11467968

[33] Evenepoel M, Daniels N, Moerkerke M, et al. Oral microbiota in autistic children: diagnosis-related differences and associations with clinical characteristics. Brain, Behav Immun - Health. 2024;38:100801. doi: 10.1016/j.bbih.2024.100801 38882715 PMC11180306

[34] Hicks SD, Uhlig R, Afshari P, et al. Oral microbiome activity in children with autism spectrum disorder. Autism Res. 2018;11(9):1286–1299. doi: 10.1002/aur.1972 30107083 PMC7775619

[35] Wingfield B, Lapsley C, McDowell A, et al. Variations in the oral microbiome are associated with depression in young adults. Sci Rep. 2021;11(1):15009. doi: 10.1038/s41598-021-94498-6 34294835 PMC8298414

[36] Forsyth A, Raslan K, Lyashenko C, et al. Children with autism spectrum disorder: pilot studies examining the salivary microbiome and implications for gut metabolism and social behavior. Hum Microbiome J. 2020;15:100066. doi: 10.1016/j.humic.2019.100066

[37] Lin D, Fu Z, Liu J, et al. Association between the oral microbiome and brain resting state connectivity in schizophrenia. Schizophr Res. 2024;270:392–402. doi: 10.1016/j.schres.2024.06.045 38986386

[38] Lee JJ, Piras E, Tamburini S, et al. Gut and oral microbiome modulate molecular and clinical markers of schizophrenia-related symptoms: A transdiagnostic, multilevel pilot study. Psychiatry Res. 2023;326:115279. doi: 10.1016/j.psychres.2023.115279 37331068 PMC10595250

[39] Shade A. Diversity is the question, not the answer. Isme J. 2017;11(1):1–6. doi: 10.1038/ismej.2016.118 27636395 PMC5421358

[40] Willis AD. Rarefaction, alpha diversity, and statistics. Front Microbiol. 2019;10:2407. doi: 10.3389/fmicb.2019.02407 31708888 PMC6819366

[41] Yucel-Lindberg T, Båge T. Inflammatory mediators in the pathogenesis of periodontitis. Expert Rev Mol Med. 2013;15:e7. doi: 10.1017/erm.2013.8 23915822

[42] Quagliato LA, Nardi AE. Cytokine alterations in panic disorder: a systematic review. J Affect Disord. 2018;228:91–96. doi: 10.1016/j.jad.2017.11.094 29241050

[43] Juruena MF, Eror F, Cleare AJ, et al. The role of early life stress in HPA axis and anxiety. Adv Exp Med Biol. 2020;1191:141–153. 32002927 10.1007/978-981-32-9705-0_9

[44] Faravelli C, Lo Sauro C, L L, et al. The role of life events and HPA axis in anxiety disorders: a review. Curr Pharm Des. 2012;18(35):5663–5674. doi: 10.2174/138161212803530907 22632471

[45] Duran-Pinedo AE, Solbiati J, Frias-Lopez J. The effect of the stress hormone cortisol on the metatranscriptome of the oral microbiome. NPJ Biofilms Microbiomes. 2018;4(1):25. doi: 10.1038/s41522-018-0068-z 30345066 PMC6194028

[46] Karkman A, Lehtimäki J, Ruokolainen L. The ecology of human microbiota: dynamics and diversity in health and disease. Ann NY Acad Sci. 2017;1399(1):78–92. doi: 10.1111/nyas.13326 28319653

[47] Liao Y, Tong X-T, Jia Y-J, et al. The effects of alcohol drinking on oral microbiota in the Chinese population. Int J Environ Res Public Health. 2022;19(9):5729. doi: 10.3390/ijerph19095729 35565124 PMC9103016

[48] Tao K, Yuan Y, Xie Q, et al. Relationship between human oral microbiome dysbiosis and neuropsychiatric diseases: an updated overview. Behav Brain Res. 2024;471:115111. doi: 10.1016/j.bbr.2024.115111 38871130

[49] Ojo-Okunola A, Claassen-Weitz S, Mwaikono KS, et al. Influence of socio-economic and psychosocial profiles on the human breast milk bacteriome of South African women. Nutrients. 2019;11(6):1390. doi: 10.3390/nu11061390 31226873 PMC6627120

[50] Ramanan P, Barreto JN, Osmon DR, et al. Rothia bacteremia: a 10-year experience at mayo clinic, Rochester, minnesota. J Clin Microbiol. 2014;52(9):3184–3189. doi: 10.1128/JCM.01270-14 24951810 PMC4313135

[51] Shen X, Li J, Li J, et al. Fecal enterotoxigenic bacteroides fragilis–peptostreptococcus stomatis–parvimonas micra biomarker for noninvasive diagnosis and prognosis of colorectal laterally spreading tumor. Front Oncol. 2021;11. doi: 10.3389/fonc.2021.661048 PMC8144651 34046355

[52] Braccia DJ, Jiang X, Pop M, et al. The capacity to produce hydrogen sulfide (H2S) via cysteine degradation is ubiquitous in the human gut microbiome. Front Microbiol. 2021;12:705583. doi: 10.3389/fmicb.2021.705583 34745023 PMC8564485

[53] Martin S, Foulon A, El Hage W, et al. Is there a link between oropharyngeal microbiome and schizophrenia? A narrative review. Int J Mol Sci. 2022;23(2):846. doi: 10.3390/ijms23020846 35055031 PMC8775665

[54] Dilek N, Papapetropoulos A, Toliver-Kinsky T, et al. Hydrogen sulfide: an endogenous regulator of the immune system. Pharmacol Res. 2020;161:105119. doi: 10.1016/j.phrs.2020.105119 32781284

[55] Ide M, Ohnishi T, Toyoshima M, et al. Excess hydrogen sulfide and polysulfides production underlies a schizophrenia pathophysiology. EMBO Mol Med. 2019;11(12):e10695. doi: 10.15252/emmm.201910695 31657521 PMC6895609

[56] Lozupone CA, Stombaugh JI, Gordon JI. Diversity, stability and resilience of the human gut microbiota. Nature. 2012;489(7415):220–230. doi: 10.1038/nature11550 22972295 PMC3577372

[57] Lenartova M, Tesinska B, Janatova T, et al. The oral microbiome in periodontal health. Front Cell Infect Microbiol. 2021;11. doi: 10.3389/fcimb.2021.629723 PMC8019927 33828997

[58] Malan-Müller S, Vidal R, O’Shea E, et al. Probing the oral-brain connection: oral microbiome patterns in a large community cohort with anxiety, depression, and trauma symptoms, and periodontal outcomes. Transl Psychiatry. 2024;14(1):419. doi: 10.1038/s41398-024-03122-4 39368974 PMC11455920

[59] Lin Y, Liang X, Li Z, et al. Omics for deciphering oral microecology. Int J Oral Sci. 2024;16(1):2. doi: 10.1038/s41368-023-00264-x 38195684 PMC10776764

[60] Song M, Bai H, Zhang P, et al. Promising applications of human-derived saliva biomarker testing in clinical diagnostics. Int J Oral Sci. 2023;15(1):2. doi: 10.1038/s41368-022-00209-w 36596771 PMC9810734

